# Optical Properties of Pyrolytic Carbon Films Versus Graphite and Graphene

**DOI:** 10.1186/s11671-015-0946-8

**Published:** 2015-05-27

**Authors:** Galyna I Dovbeshko, Volodymyr R Romanyuk, Denys V Pidgirnyi, Vsevolod V Cherepanov, Eugene O Andreev, Vadim M Levin, Polina P Kuzhir, Tommi Kaplas, Yuri P Svirko

**Affiliations:** Institute of Physics of NAS of Ukraine, Prospect Nauki 46, Kyiv, 03680 Ukraine; V. Lashkaryov Institute of Semiconductor Physics NAS of Ukraine, Prospect Nauki 41, Kyiv, 03680 Ukraine; Institute of Biochemical Physics, RAS, Moscow, Russia; Research Institute for Nuclear Problems of Belarusian State University, Bobruiskaya Str. 11, Minsk, 220030 Belarus; Ryazan State Radio Engineering University, 59/1 Gagarina Street, Ryazan, 390005 Russia; Institute of Photonics, University of Eastern Finland, Yliopistokatu 7, P.O. Box 111, Joensuu, FI-80101 Finland

**Keywords:** 78.66.Db, 78.20.Ci, 81.05.U-, Pyrolytic carbon films, Graphite, Graphene layers, Optical properties

## Abstract

We report a comparative study of optical properties of 5–20 nm thick pyrolytic carbon (PyC) films, graphite, and graphene. The complex dielectric permittivity of PyC is obtained by measuring polarization-sensitive reflectance and transmittance spectra of the PyC films deposited on silica substrate. The Lorentz-Drude model describes well the general features of the optical properties of PyC from 360 to 1100 nm. By comparing the obtained results with literature data for graphene and highly ordered pyrolytic graphite, we found that in the visible spectral range, the effective dielectric permittivity of the ultrathin PyC films are comparable with those of graphite and graphene.

## Background

Carbon can exist in a number of allotropic forms including diamond, graphite, graphene, fullerenes, and carbon nanotubes (CNT) that also have a great deal of variability. The carbonaceous materials are recently attracting ever growing attention of the research community due to their strong potential in electronics, optics, medicine, etc. However, among planar carbon materials with *sp*^2^ hybridization of electron orbitals, the researchers were mainly focused on highly ordered pyrolytic graphite (HOPG) and graphene. HOPG is a highly ordered synthetic bulk material, which is characterized by strong anisotropy of optical, electronic, and elastic properties [1–3]. Graphene is a two-dimensional carbon material comprising a one-atom thick layer of graphite. Optical and electronic properties of graphene are determined by zero bandgap and linear dependence of the electron energy on momentum in the vicinity of K-point of Brillouin zone. This in particular results in the nearly featureless visible absorption spectrum of graphene, which absorbs 2.3 % of incident radiation in the wavelength region of 350–800 nm [4, 5].

**Fig. 1:**
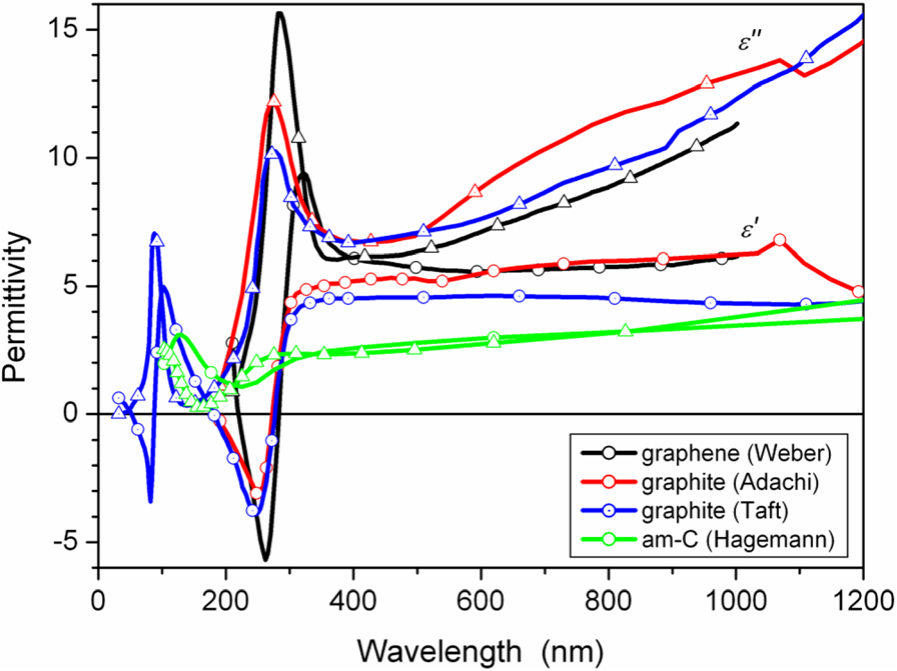
Real (*circles*) and imaginary (*triangles*) parts of complex dielectric permittivity of graphite and graphene according to [1, 3, 5] and amorphous carbon [10]

Much less attention was paid to the electronic and optical properties of nanometrically thick pyrolytic carbon (PyC) films consisting of disordered and intertwined graphene flakes with the linear size of several nanometers [6–8]. Biocompatibility, durability, wear resistance, and robustness make this carbon material attractive for bio- and medical applications [9]. However, the optical and electronic properties of these films may be influenced by the synthesis conditions [6–13]. That is why a comparative study of the graphene-based materials with short-range (e.g., PyC) and long-range (e.g., graphene) crystalline ordering may provide deeper insight on the synthesis and physical mechanisms responsible for the properties of carbon materials with dominating *sp*^2^ hybridization of electron orbitals. Such a study should include a detailed analysis of the strong absorption band near 240–300 nm (Fig. 1), which originates from the π–π^∗^ electron transition and is observed in single- and multilayer graphene [5], carbon nanotubes [11], and HOPG [1, 3]. This band is of special interest because of recent results on the application of carbon films for surface-enhanced Raman scattering (SERS), surface-enhanced infrared absorption (SEIRA) [12, 13], and surface-enhanced coherent anti-stokes Raman spectroscopy (CARS) [14]. It was surprising that PyC and HOPG substrates do not provide enhancement in SERS, while a single-walled CNT substrate does [13]. At the same time, graphene and CNT substrates provide a strong enhancement for surface-enhanced CARS [14–16]. These experimental findings are still not fully understood and require a comprehensive and comparative analysis of optical and electronic properties of these *sp*^2^ materials.

In this paper, we compare optical properties of ultrathin PyC films, few graphene layer thick films, and HOPG. The paper is organized as follows. Experimental details and samples fabrication methods are discussed in the “Methods” section. The “Results and Discussion” section describes experimental data and results of comparative analysis of dielectric properties along with the modeling of PyC film optical properties. The conclusions are outlined in the “Conclusions” section.

## Methods

In our experiments, the PyC films were synthesized on the 0.5 mm thick silica substrate by chemical vapor deposition (CVD), described in more detail elsewhere [6]. Briefly, the process occurs in the CVD chamber, which is initially heated up to 700 °C in hydrogen atmosphere (10 mbar). Then, the hydrogen atmosphere is replaced by CH_4_–H_2_ gas mixture. The methane decomposition takes place when the chamber was heated up to 1100 °C during 5 min and kept under this temperature during several minutes and then again cooled down to 700 °C. At the temperature of 700 °C, the CH_4_–H_2_ gas mixture was replaced by hydrogen. Film thickness depends on methane concentration in CVD chamber. In the CVD process, both sides of the silica substrate were covered by the film. For optical transmission and reflection measurements, one side of the substrate was cleaned by oxygen plasma. In this paper, we study PyC films with thickness of 5, 8, 14, and 20 nm.

Surface morphology of the deposited films was studied by the Solver Pro M (NT-MDT) atomic force microscope in taping mode with NSG10 atomic force microscopy (AFM) probes. In order to determine surface profile parameters, we applied the deconvolution algorithm procedure [17] to the obtained AFM data.

Optical measurements were performed in the 360–1100 nm wavelength range with a DMR-4 (LOMO, USSR) spectrophotometer equipped with incandescent lamp as a light source. Reflectance, *R*_p_(λ), and transmittance, *T*_p_(λ), spectra were recorded for *p*-polarized light at several angles of light incidence using spectral bandwidth of 3 nm. The beam spot diameter on the sample surface was about 3 mm.

Complex dielectric permittivity ε *=* ε*′ + i*ε*″* (ε*′ = n*^*2*^ *− k*^*2*^, ε*″ = 2nk*, where *n* is the refractive index and *k* is the extinction coefficient) of the PyC films were obtained from the simultaneous fitting of measured transmitted and reflectance spectra with those calculated with 2 × 2 matrix formalism [18, 19] for a “homogeneous isotropic film on the substrate” structure. The dependence of the complex dielectric permittivity on the photon energy in the spectral range of interest was approximated by using the Lorentz-Drude model.

## Results and Discussion

**Fig. 2:**
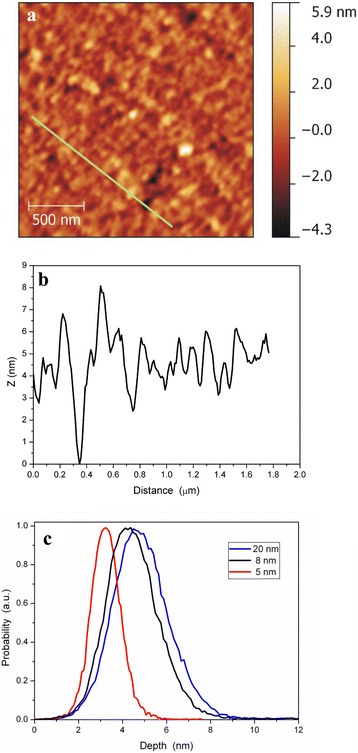
2D AFM image (**a**), Z-profile along the line marked on the image of 5 nm thick PyC film (**b**), and statistical analysis of profile height distribution (bearing analysis) for 5, 8, and 20 nm thick PyC film samples (**c**)

The morphology, the grain size, and the surface roughness of all deposited films were obtained using AFM. Fig. 2a shows AFM image of the 5 nm thick PyC film. One can observe that the film has a granular structure with correlation length of surface roughness less than 100 nm. The transmittance electron microscopy (TEM) measurements [20] showed that the film consists of the randomly oriented and intertwined graphene flakes with linear size of 5–10 nm with amorphous carbon inclusions. The presence of randomly oriented graphene flakes results in the surface roughness, which is not changed considerably with the film thickness. That is, the root mean square (*rms*) surface roughness is 1.2–1.5 nm for all studied PyC films. Analysis of the surface relief also revealed the presence of nanopores (voids). One can see from Fig. 2a and b that the thinnest film contains a small fraction of pores. Statistical distributions of film surface profile heights for 5 and 8 nm thick PyC films have the total width comparable with the film thickness (Fig. 2c). It means that pores penetrate through entire film thickness. However, in the thickest film, the width of the surface profile height distribution is much smaller than the average film thickness of 20 nm. These voids and surface roughness should be taken into account when the PyC films are characterized by the optical transmission/reflection measurements.

**Fig. 3:**
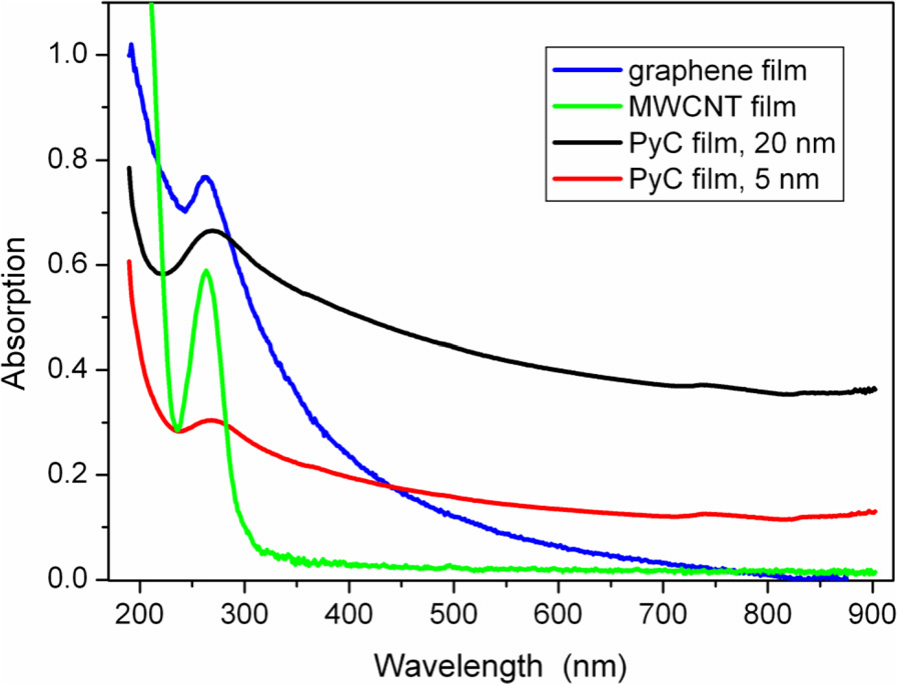
UV–vis absorption spectra of some carbonaceous planar materials with *sp* ^2^ hybridization of electron orbitals: graphene 3-layer thick film, suspended MWCNT, 5 and 20 nm thick PyC films

Absorption spectra of PyC, graphene, and multi-walled CNT films on silica substrate are presented in Fig. 3. One can observe that the absorption spectra of all three materials are dominated by π–π^∗^ electronic transitions at photon energy (wavelength) of ~4.6 eV (~270 nm). This absorption resonance is a signature of *sp*^2^ hybridized electronic orbitals. No such a resonance is observed in the absorption spectra of amorphous carbon, diamond, and diamond-like carbon [21, 22]. It is worth noting that π–π^∗^ absorption band originates from interband transitions, while graphene and graphite possesses nearly zero bandgap [21–24]. Similar to graphene and CNT, PyC films have the absorption band centered at 270 nm which originates from *sp*^2^ hybridization of electron orbitals. One can observe from Fig. 3 that this absorption resonance is broader than that in a few layered graphene. This broadening can be explained by the disordered and intertwined graphene flakes and presence of amorphous carbon. In particular, since PyC film consists of nanosized graphene flakes, the band structure of each flake in the vicinity of the M-saddle point depends on the flake size, shape, and orientation. This makes the resonance less pronounced than that in graphene and carbon nanotubes. Moreover, the intensive electron scattering on the flake boundaries and grains of the amorphous carbon broadens the absorption resonance at 270 nm even further.

**Fig. 4:**
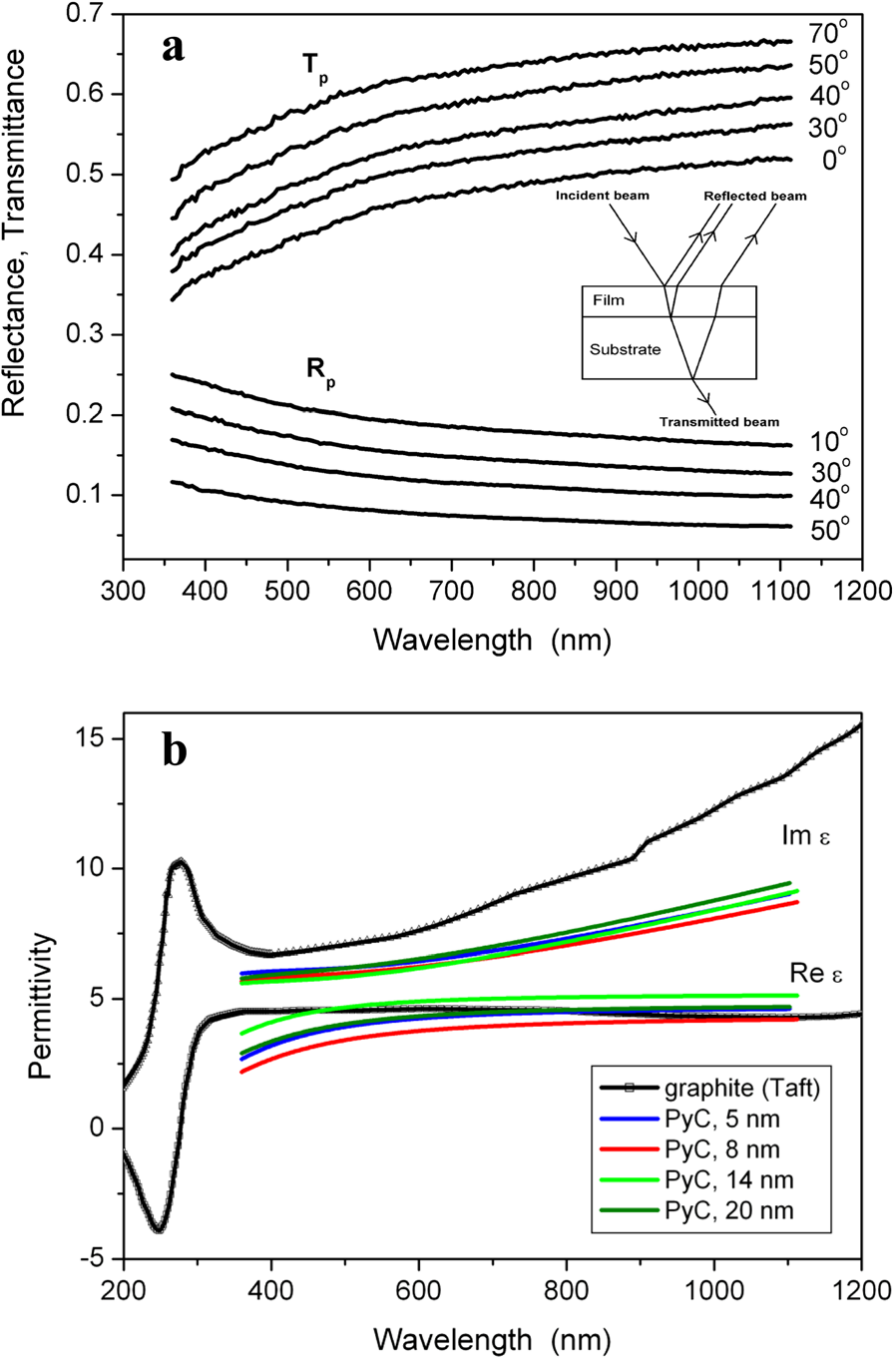
Experimental reflectance and transmittance spectra of 14 nm thick PyC film for *p*-polarized light at several angles of incidence (**a**), and optical parameters of pyrocarbon (**b**)

In order to obtain wavelength dependence of the optical parameters of the PyC films, we measured simultaneously both transmittance and reflectance spectra for the *p*-polarized incident light beam. Fig. 4a presents experimental transmittance and reflectance spectra of 14 nm PyC film in the wavelength range 360–1100 nm. One can observe that both transmittance and reflectance show strong wavelength dependence in the blue part of the studied spectral range, i.e., in the vicinity of π–π^∗^ resonance at 270 nm. It is worth mentioning that correlation length of surface roughness is about 100 nm, and hence, the scattered light may influence measured transmission and reflection coefficients. However, since the light scattering mainly affects reflection [25], we performed additionally transmittance measurements at several angles of light incidence and recover the optical properties of the PyC films from transmittance and reflectance measured at several angles of incidence.

In the UV range, energy dispersion of the complex dielectric permittivity ε(*E*) of layered carbonaceous materials (see Fig. 1) dominate by two single-electron resonances, which are centered at ~85 and ~270 nm and correspond to σ–σ^∗^ and π–π^∗^ transitions, respectively, while at the visible and IR wavelengths, ε is determined by free electron gas. This allows us to employ the combined Lorentz-Drude model, which is consistent with the Kramers–Kronig relations, to describe the optical properties of PyC films. This model is conventionally used to describe properties of metals (e.g., silver) in the spectral range where the both free carriers and bound electrons (e.g., *d*-electrons in silver) contribute to the dielectric constant.

For carbon materials with dominating *sp*^2^ bonds, the Lorentz-Drude model yields:

1 $$ \varepsilon (E)={\varepsilon}_{\infty }-{\varepsilon}_{\infty}\frac{{E_{pl}}^2}{E^2+iE{\gamma}_{pl}}+\frac{A{E_T}^2}{{E_T}^2-{E}^2-iE{\gamma}_T} $$

where the ε_∞_ is the high frequency dielectric constant; *A*, *E*_T_, and γ_T_ are the strength, resonant energy, and FWHM of the π–π^∗^ electron transition; and *E*_pl_ and γ_pl_ are the plasma energy and free electron dephasing rate. Since we measured reflection and transmission in the wavelength range 360–1100 nm, we take into account only one resonance at *E*_T_ = 4.6 eV (270 nm) that represents π–π^∗^ electron transition. Five parameters (ε_∞_, *A*, γ_T_, *E*_pl_, and γ_pl_) that determine dispersion of the dielectric permittivity in Eq. (1) should be determined from fitting of the reflectivity and transmittance spectra measured for several light-incidence angles. The thickness of the studied PyC films (i.e., 5 ± 0.7, 8 ± 0.9, 14 ± 1.2, and 20 ± 2 nm) was determined by TEM microscopy and by a stylus profiler (Veeco Instruments, Dektak 150). The results of the fitting of the measured spectra are presented in Table 1. One can observe that fitting parameters including plasma frequency vary due to thickness uncertainty and so-called sample effect [26, 27]. Particularly, plasma frequencies slightly differ and are found near to 10 eV.

**Table 1:** Parameters of Lorentz-Drude approximation for PyC permittivity spectral dependencies in the 350–1100 nm spectral region

PyC film thickness (nm)	ε_∞_	*A*	γ_T_ (eV)	*E*_pl_ (eV)	γ_pl_ (eV)
5	1.38	3.783	5.36	11.39	23
8	1.37	3.233	5.60	10.42	21
14	2.35	3.082	5.36	8.08	24
20	1.83	3.442	5.46	10.19	20

Figure 4b presents wavelength dependence of the real (ε*′*) and imaginary (ε*″*) parts of the complex effective [24] permittivity of PyC films deposited on the silica substrate calculated by using Eq. (1) with parameters presented in Table 1. One can observe that obtained ε*′* and ε*″* of the PyC films in the spectral range 360–1100 nm are comparable with those of graphite [1]. Graphite as a semimetal with a uniaxial-layered crystalline structure and hence anisotropic permittivity possesses dielectric properties along the orientation normal to the graphene layer and metallic conductance along to the graphene layer. The measurements of graphite permittivity tensor components in [28] indicate that the real part of the transverse graphite permittivity (ε_⊥_ ') is positive, whereas the parallel component of graphite permittivity (ε_||_ ') is negative at ultraviolet wavelengths below 282 nm. Our measurements on isotropic PyC show that in the spectral range 360–1100 nm ε*′* >0 and ∣ε*′*/ε*″*∣ < 1.

It is known [29] that ε*′* < 0 and ∣ε*′*/ε*″*∣ > 1 are the main conditions for electromagnetic field localization and enhancement in plasmonic structures. When these conditions are fulfilled, the structures can be used as substrates in SEIRA and SERS, in which the signal enhancement are ~ ∣ε*′*/ε*″*∣^2^ and ~ ∣ε*′*/ε*″*∣^4^, respectively. In graphite, π + σ-plasmon is located near 15 eV [30], while in graphene [31] and carbon nanotubes [32], it is in the terahertz region. This implies that these carbon materials cannot be employed as substrates for surface-enhanced spectroscopy with excitation at the visible wavelengths. However, one may expect that conditions ε*′* < 0 and ∣ε*′*/ε*″*∣ > 1 can be fulfilled in the ultraviolet region for PyC. Therefore, further investigation of the frequency dispersion of the PyC permittivity in UV region, especially below 300 nm, might be interesting for surface-enhanced spectroscopy applications.

It is also important that despite variation of fitting parameters in Eq. (1) and film thickness, fitting produces nearly the same ε*′* and ε*″* for all films. This indicates that the Lorentz-Drude model describes well the general features of the optical properties of PyC. It is worth noting that the variation of the real and imaginary parts of the permittivity of PyC films can be explained in terms of the effective medium approximation if one takes into account the thickness of surface rough overlayer and small voids content as obtained from AFM investigations.

## Conclusions

In this work, the optical properties of pyrolytic carbon films in the visible spectral range have been studied with reflectance/transmittance spectroscopy. We have shown that dielectric permittivity of the PyC films deposited on silica substrate are quite similar to those obtained for graphite and graphene. Thus, the real part of PyC permittivity, ε*′*, is positive, and the ratio of the real and imaginary part of dielectric permittivity ∣ε*′*/ε*″*∣ for PyC in the visible spectral range is less than 1, being a condition of absence of electromagnetic enhancement in SERS for molecules absorbed on PyC films exited by visible light. We demonstrate that the Lorentz-Drude model describes well the general features of the optical properties of PyC from 360 to 1100 nm. However, atomic force and transmission electron microscopy investigations have shown that films possess a granular morphology and rough surface that should be taken into account in future experiments.

## Abbreviations

AFM: atomic force microscopy
CARS: coherent anti-stocks Raman scattering
CNT: carbon nanotubes
CVD: chemical vapor deposition
DLC: diamond-like Carbon
ε: dielectric permittivity
HOPG: highly ordered pyrolytic graphite
n: refraction index
k: extinction coefficient
PyC: pyrolytic carbon
SEIRA: surface-enhanced infrared absorption
SERS: surface-enhanced Raman scattering
TEM: transmission electron microscopy
